# Relative Importance of Biotic and Abiotic Soil Components to Plant Growth and Insect Herbivore Population Dynamics

**DOI:** 10.1371/journal.pone.0012937

**Published:** 2010-09-23

**Authors:** Martijn L. Vandegehuchte, Eduardo de la Peña, Dries Bonte

**Affiliations:** Department Biology, Ghent University, Ghent, Belgium; Institut Mediterrani d'Estudis Avançats (CSIC/UIB), Spain

## Abstract

**Background:**

Plants are affected by several aspects of the soil, which have the potential to exert cascading effects on the performance of herbivorous insects. The effects of biotic and abiotic soil characteristics have however mostly been investigated in isolation, leaving their relative importance largely unexplored. Such is the case for the dune grass *Ammophila,* whose decline under decreasing sand accretion is argued to be caused by either biotic or abiotic soil properties.

**Methodology/Principal Findings:**

By manipulating dune soils from three different regions, we decoupled the contributions of region, the abiotic and biotic soil component to the variation in characteristics of *Ammophila arenaria* seedlings and *Schizaphis rufula* aphid populations. Root mass fraction and total dry biomass of plants were affected by soil biota, although the latter effect was not consistent across regions. None of the measured plant properties were significantly affected by the abiotic soil component. Aphid population characteristics all differed between regions, irrespective of whether soil biota were present or absent. Hence these effects were due to differences in abiotic soil properties between regions. Although several chemical properties of the soil mixtures were measured, none of these were consistent with results for plant or aphid traits.

**Conclusions/Significance:**

Plants were affected more strongly by soil biota than by abiotic soil properties, whereas the opposite was true for aphids. Our results thus demonstrate that the relative importance of the abiotic and biotic component of soils can differ for plants and their herbivores. The fact that not all effects of soil properties could be detected across regions moreover emphasizes the need for spatial replication in order to make sound conclusions about the generality of aboveground-belowground interactions.

## Introduction

Plants are heavily affected by both the abiotic and biotic properties of the soil in which they are rooted. Abiotic properties include the availability of nutrients and water, which are necessary for plant growth. Soil biota comprise mutualists as well as antagonists, exerting positive or negative effects on plant growth respectively.

These effects of soil properties on the plant can further affect leaf herbivores. Firstly, herbivores are generally limited by the nutrients they can obtain from plants, some of which these plants extract from soil [1], such as nitrogen [2], [3] and phosphorus [4]. Secondly, soil biota can exert positive, negative or neutral effects on leaf herbivores, through a variety of mechanisms (reviewed in [5]–[10]). Positive effects can for instance arise when soil biota improve the nutritional quality of leaves [11]–[13], or damage production sites of defence molecules in the roots [14]. Most negative effects of soil biota on leaf herbivores have been attributed to the systemic induction of chemical defences that spread from roots to shoots [15], although root-feeders can also cause a lowering of amino acid levels in leaves [16].

Although both biotic and abiotic soil components clearly have the potential to affect the performance of plants and their herbivores, their relative contribution to these effects has rarely been addressed. In a cross-inoculation experiment of soil biota, Joosten et al. [17] demonstrated that both soil-borne microorganisms and the type of sterile soil affected dry mass, shoot/root ratio and the total amount and composition of pyrrolizidine alkaloids in *Jacobaea vulgaris*. As these compounds are toxic to generalist herbivores, but preferred by specialists, it is concluded that both the abiotic and biotic soil component have the potential to affect herbivores of *J. vulgaris*. To our knowledge only a few studies have explicitly tested the combined effect of abiotic and biotic soil components on aboveground herbivores. Haase et al. [18] investigated the interactions between collembolans (*Folsomia candida*) and aphids (*Rhopalosiphum padi*) on the grass *Poa annua* under different levels of nutrient availability. They demonstrated that collembolans strongly increased aphid numbers at low and moderate nutrient availability, while this effect was much weaker at high nutrient availability. It has furthermore been demonstrated that the effect of mycorrhizal fungi on the performance of an insect can depend on the amount of nutrients in the soil [9], especially P [12], [13] and N [19]. The use of artificial fertiliser and the addition of large quantities of soil organisms in most of these studies raise the question whether these findings can be extrapolated to the field. Moreover, none of these studies accounted for the potential spatial variation in nutrient availability and/or soil organism density and identity one might expect to occur in natural ecosystems.

One of the best studied and most debated cases of negative plant-soil feedback is that of *Ammophila* species in the early succession of coastal dune vegetation. Both the North American *A. breviligulata* and the European *A. arenaria* exhibit strongly suppressed growth as sand accretion ceases, making way for later successional plant species. Therefore, vigorous stands of *Ammophila* are found in foredunes and large, dynamic inland dunes with sufficient sand-drift, while stands in stabilised dunes, often at the inner dune edge, occur as degenerate relics. This phenomenon of loss of vigour under conditions of stabilisation has been coined “the *Ammophila* problem” by Marshall [20]. The earlier work proposed interspecific competition with other plant species [21], [22] and lack of nutrients because of an inefficient replacement of old roots [20], [23] as explanations for the problem. However, further study on *A. arenaria* revealed that the accumulation of biotic soil factors in stabilised soils was responsible for reduced growth [24]–[26]. The windblown sand would thus serve as a temporary enemy-free space for the plant to root in, this is the so called “escape hypothesis”. Continued investigation along this line led to the conclusion that both plant-parasitic nematodes and pathogenic fungi might be the causing agents of the observed decline [27]–[29]. The effect of antagonistic nematodes on plant performance was shown to be mitigated by the positive influence of mycorrhizae [30], [31] and endophytic fungi [32]. The outcompeting of harmful nematodes by less detrimental nematode species furthermore proved to be beneficial for the plant [33], [34], but see Brinkman et al. [35]. Moreover, it was shown that different species of root-feeding nematodes can be controlled in specific ways by soil microorganisms, other nematodes and microarthropods [36], [37]. Although these studies point at the complex nature of the interactions between different soil biota involved in this system, their net effect seems to be negative, indicating that mutualists are generally not able to overcome the effects of antagonists [25], [37]. In recent years, the original hypothesis of root efficiency of nutrient uptake has revived [38]. A study by Kooijman et al. [39] furthermore attributes the expansion of *A. arenaria* in lime- and iron-poor dunes to the limitation by N as a result of the relatively higher availability of P when it is not sequestered into iron and aluminium phosphates.

In a previous study, we demonstrated that the natural root-feeding nematode community of *A. arenaria* was capable of reducing the population growth of the specialist aphid *Schizaphis rufula* under laboratory conditions. Yet no correlation between nematode and aphid abundances could be detected in a field survey conducted at six spatially separated sites. Both nematode and aphid abundances could however be explained by several plant characteristics [7]. This suggests that the effect of nematodes on aphids might be overruled in the field by other environmental factors with stronger effects on those plant traits that determine the aphids' performance.

Given this multitude of studies using *A. arenaria* to investigate plant-soil interactions, we chose this species as a model system. Based on the evidence that soil-borne organisms are involved in the *Ammophila*-problem, we hypothesise that plants should grow better on sterile soils compared to soils with naturally occurring biota. On the other hand, if this phenomenon is to some extent caused by an increased availability of nutrients in dynamic dune soils, plants should perform better on dynamic than on stabilised dune soils, irrespective of their biotic state. We inoculated sterile soils from dynamic and stabilised dunes with biota from either location and used a fully sterile soil as control. On these soils we grew seedlings of *A. arenaria*, on half of which we let a population of *S. rufula* develop. This setup was replicated with soils from three distinct regions along the Belgian coast, to assess the generality of potential results across large spatial scales [40]. This fully-crossed experiment allowed us to specifically address the following questions: 1) what is the relative importance of biotic and abiotic soil properties regarding their effect on aphid population dynamics? 2) are potential effects consistent across spatially separated dune systems, or are there regional differences? 3) if abiotic soil properties are important, which ones would make plausible candidates to explain the observed effects? We hypothesise that the lower availability of nutrients should cause the performance of aphids on plants grown in sterile soils to be lower for stabilised than for dynamic dune soils. Because the accumulation of plant pathogens in stabilised dune soils is expected to result in reduced plant growth, we hypothesise that aphids should perform worse on soils inoculated with stabilised dune biota. Given the usual variability in the distribution of soil nutrients and organisms, we expect the magnitude of these effects to differ between regions. We hypothesise that the availability of nitrogen and phosphorus in soils should match the performance of aphids better than that of other abiotic elements.

## Materials and Methods

### Experimental setup

Soil was collected from three different regions at the coast on 5 November 2008: nature reserve Westhoek at De Panne (Belgium), nature reserve Ter Yde at Oostduinkerke (Belgium) and Le Perroquet at Bray-Dunes (France). In each region soil was collected from two sites; one situated in dynamic dunes with sand-drifts where *A. arenaria* grows very vigorously and one situated at the inner dune edge, where conditions are more stabilised and the plant only occurs as degenerate relics. All these dune areas are spatially separated (distances between sites from different regions ranging from 2.1 to 12.6 km). At each site, a composed sample of soil was taken, collected from underneath different stands of *A. arenaria,* comprising a mixture of upper root zone soil and freshly deposited soil from above the root zone. In the laboratory each sample of soil was divided into two parts, one of which was sterilised by autoclaving for 1 hour at 120°C and 1 atm. For each site three types of soil were prepared: fully sterile soil, sterile soil with an inoculum of unsterile soil from the same site and sterile soil with an inoculum of unsterile soil from the other site within the same region. So for each region the combinations were: D, D+s , D+d, S, S+d, S+s, with D  =  sterile dynamic dune soil, S  =  sterile stabilised dune soil, d  =  unsterilised dynamic dune soil inoculum, s  =  unsterilised stabilised dune soil inoculum. The inoculum comprised 21 volume percent of the soil mixture, which did not affect any of the purely abiotic properties of the soil mixture (see Text S1, Fig. S1, Fig. S2, Fig. S3, Table S1 and Table S2). This way the effect of the soil's abiotic and biotic component could be decoupled.

Seeds of *A. arenaria* were collected from the nature reserve Westhoek from a single stand. Seeds were surface sterilised by submersing in 4% household bleach solution, rinsing 10 times with demineralised water, submersing in 10% ethanol and rinsing another 10 times with demineralised water. This sterilisation method effectively eliminates endophytic fungi that otherwise could colonise the young seedling. Seeds were subsequently germinated at a light regime of 9/15 hours dark/light in plastic 1 L pots filled with 190 cm^3^ of commercial white sand that was autoclaved for 1 hour at 120°C and 1 atm. The sand was saturated with demineralised water. Plastic foil that covered the pots was perforated to allow of enough ventilation. Moisture level was reset to near saturation 3 times a week.

For each unique soil type, twenty replicate 1062 mL glass jars were each filled with 300 mL of the treatment soil. Each jar received one six week old seedling, and from then on water was added twice a week, alternately with and without fertiliser (Compo NPK 16-9-20, 1 g L^−1^ tap water).

After having grown on the different soils for 5 weeks, ten plants of each treatment soil type received a single first instar nymph of the specialist aphid *S. rufula*, that was allowed to become adult and reproduce parthenogenetically. Aphids were counted daily. When a substantial decrease in aphid numbers occurred, the plant was harvested and all remaining aphids were transferred to 70% ethanol. The plant was uprooted and root and shoot were weighed fresh. They were subsequently oven dried at 65°C overnight and weighed again.

Plants that did not receive aphids acted as a control. They were uprooted 12 weeks after transplantation to the different soil types. Root and shoot were weighed fresh before they were oven dried at 65°C overnight to determine dry weights. Of each soil type three replicates were selected for soil analysis. The following soil characteristics were determined: percentage moisture (g water per 100 g of fresh soil), NO_3_-N (mg kg^−1^ of dry soil), NH_4_-N (mg kg^−1^ of dry soil), plant available P (mg kg^−1^, Olsen method), pH-KCl, percentage organic matter per dry soil and percentage CaCO_3_ (see Text S1 for methodology).

### Analyses

The effects of the soil treatments on plant performance were tested within the control group of plants that did not receive aphids, because the effect of aphids on plants can potentially interact with the effects of the soil treatments. Tested plant variables were total dry weight, the root proportion of total dry weight and relative water content of the shoot, which was highly correlated with the relative water content of the entire plant. These variables were chosen because they represent a measure of total biomass produced, the relative allocation of biomass to roots and shoots, and the vitality of the plant tissue respectively.

Several aphid population parameters were tested in function of the soil treatments. The number of days between introduction of the first instar and the appearance of the first offspring was used as an approximation of generation time. The number of aphids at the population peak equates the maximum population size a plant can sustain. For each population, an exponential growth curve was fitted through the aphid abundances from day one until te day of population peak. The growth constant k of the curve N  =  N_0_.e^kt^ served as a measure of population growth speed.

To determine which treatments or interactions between treatments significantly affected plant and aphid characteristics, a permutational 3×2×3 ANOVA was performed for each dependent variable. The treatment “region” was composed of levels “Westhoek” (WE), “Ter Yde” (TY) and “Perroquet” (PE). The treatment “soil” refers to the sterile part of each treatment soil with levels “dynamic dune” (D) and “stabilised dune” (S). The treatment “inoculum” refers to the unsterile soil inoculum with levels “none” (/), “dynamic dune” (d) and “stabilised dune” (s). Tests were based on type III sums of squares and 99.999 permutations of the residuals under a reduced model. A backward stepwise pooling of non significant terms (*P*>0.05) was performed to obtain robust *P*-values in the final model. If the final model happened to retain only one predictor variable, the test was repeated with unrestricted permutation of raw data, which provides an exact test for the one-way case [41]. Homogeneity of variances was tested with a permutational Levene's test using 99.999 permutations. In the case of unequal variances, a non-parametric test was performed by applying the above-described permutational ANOVA to the ranks of the original data. Pairwise differences between levels of significant factors were tested for each final model by means of a permutational t-test with 99.999 permutations. When the number of unique permutations was lower than 100, Monte Carlo sampling was used to obtain reliable *P*-values.

Correlations between each aphid population parameter and the different plant characteristics were determined across all replicas that had received aphids. The plant feature that correlated best with each dependent variable was incorporated as a continuous covariate into the above-described ANOVA models. By comparing models with and without covariate, effects of soil treatments on aphids that are due to differences in plant growth or vitality could be decoupled from additional plant physiological effects.

Out of 180 introduced aphid nymphs, 16 died before reproducing and these replicas were omitted from all analyses. Seedlings that died in the early stages after transplantation (77 out of 360) were replaced, but since this replacement affected the measured characteristics, only the initial plants were retained in all analyses. There were no significant differences in seedling mortality between regions, soil types, or inocula (Vandegehuchte et al. unpub. data).

To determine whether the soil properties of our treatment soils significantly differed according to region, soil, inoculum, or an interaction, a permutational 3×2×3 ANOVA with a backward selection procedure was performed for each soil parameter in a similar way as described above.

## Results

### Plants

**Figure 1:**
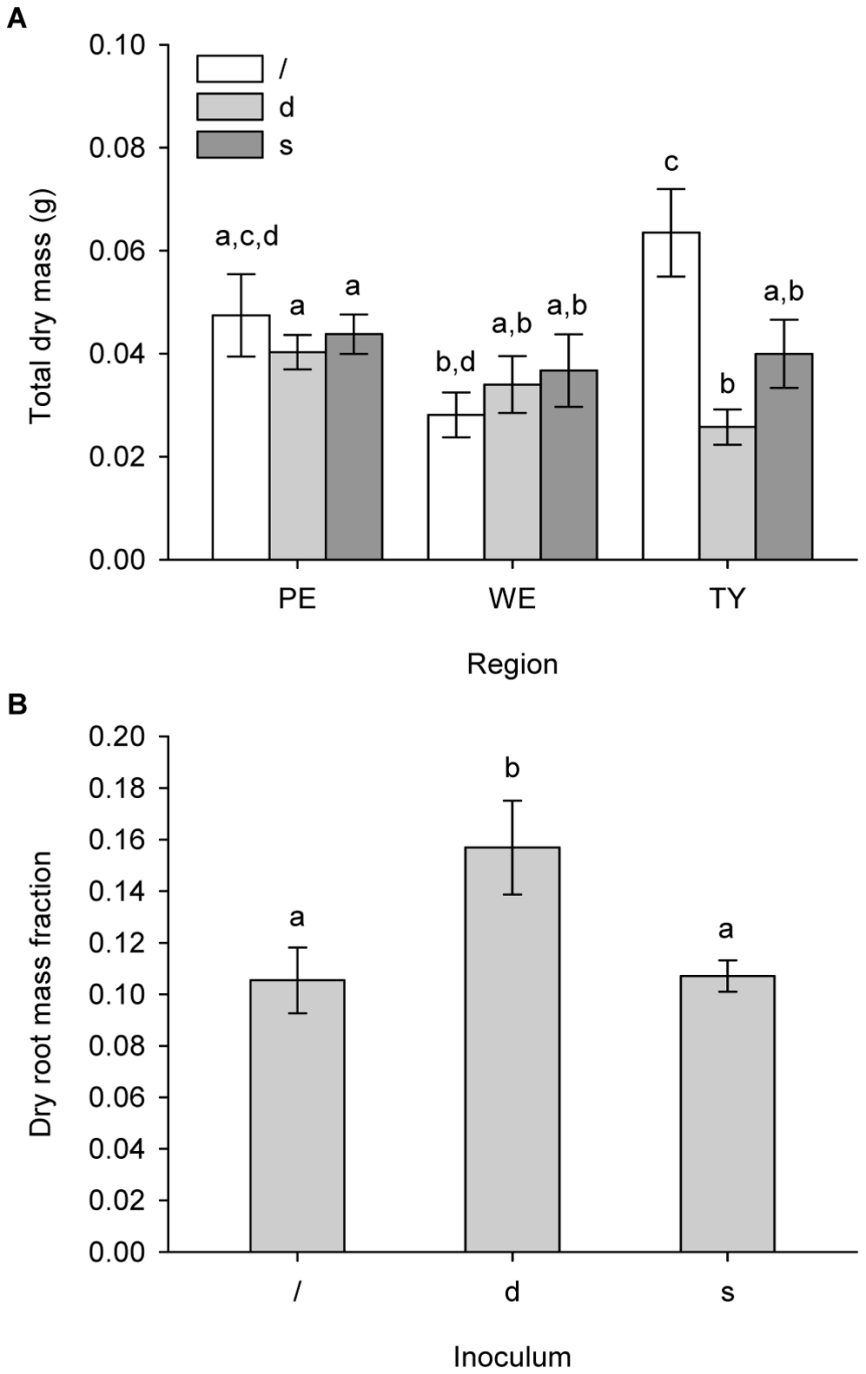
Effect of different soil treatments on characteristics of *A. arenaria* seedlings (mean + SE). A) Effect of region and soil inoculum on total dry mass of the plant. B) Effect of soil inoculum on the root fraction of the total plant dry mass. Significant pairwise differences are indicated by different letters above the bars (*P*<0.05). Region - PE: Le Perroquet, WE: Westhoek, TY: Ter Yde. Inoculum -/: no inoculum, d: dynamic dune biota, s: stabilised dune biota.

Plant dry weight differed significantly according to an interaction between region and inoculum (pseudo-F_4,93_: 3.7674, *P*: 0.0068, Fig. 1), with the main effect of inoculum being significant (pseudo-F_2,93_: 3.4922, *P*: 0.0336) and the main effect of region being marginally significant (pseudo-F_2,93_: 2.7043, *P*: 0.0711). The type of sterile soil did not affect plant dry weight. Pairwise comparisons revealed that on soils from Westhoek and Le Perroquet there was no effect of soil biota on total dry weight, regardless of their origin. On Ter Yde soils, however, dry weight clearly increased when no soil biota were inoculated (Table S3).

Although for plant dry weight the Levene's test was significant (*P*: 0.0269), the F-test of ANOVA is very robust against unequal variances, and a significance level of 0.01 has been suggested for homogeneity tests prior to ANOVA [42], [43]. We therefore consider the presented results valid. The non-parametric test moreover confirmed the significant interaction between region and inoculum (pseudo-F_4,93_: 3.2103, *P*: 0.0165).

Homogeneity of variances could not be confirmed for the relative water content of the shoot (Levene's test, *P*: 0.001), and according to the non-parametric test, none of the treatments had a significant effect.

The root fraction of dry weight was only affected by soil inoculum (pseudo-F_2,99_: 4.8511 , *P*: 0.0025), as a larger proportion of the total dry weight was allocated to roots in plants grown on soils inoculated with biota from dynamic dunes (Fig. 1). This effect was irrespective of whether the sterile soil part originated from dynamic or stabilised dunes and of the region along the coast. Variances were homogeneous according to Levene's test (*P*: 0.3768). Summarising these results, it can be concluded that *A. arenaria* seedlings were most affected by soil biota.

### Aphids

The plant feature that correlated best with aphid maximum density was the fresh weight of the shoot (Pearson's r: 0.74221). Total fresh weight correlated best with generation time (Pearson's r: 0.49349), while the exponential growth constant correlated best with relative water content of the shoot (Pearson's r: 0.32240).

**Figure 2:**
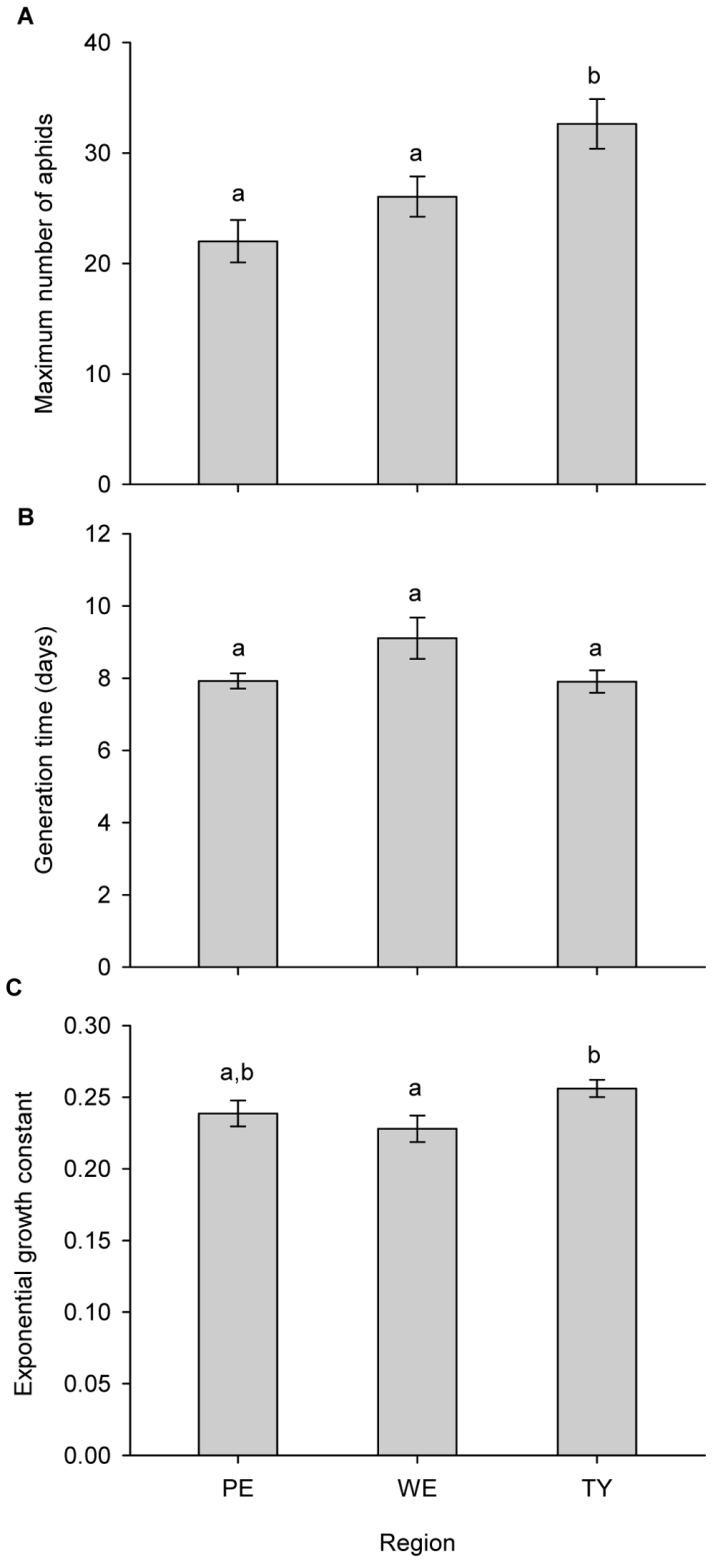
Effect of soil region of origin on *S. rufula* aphid population dynamics (mean + SE). A) Effect of region on the maximum number of aphids. B) Effect of region on the generation time of the first aphid. C) Effect of region on the growth constant k of the exponential growth curve. Significant pairwise differences are indicated by different letters above the bars (*P*<0.05). Region - PE: Le Perroquet, WE: Westhoek, TY: Ter Yde.

Results of the ANOVA demonstrate a significant effect on maximum aphid density only of region (pseudo-F_2,161_: 7.174, *P*: 0.001, Fig. 2). Modelling shoot fresh weight as a covariate (pseudo-F_1,160_: 194.88, *P*: 0.00001) still resulted in a significant effect of region (pseudo-F_2,160_: 6.7923, *P*: 0.0008). This indicates that regional soil effects do not only operate through changes in plant shoot weight, but also through additional mechanisms. Again only region was retained as a significant factor affecting aphid generation time, both in the models without (pseudo-F_2,161_: 3.1168, *P*: 0.0356) and with (pseudo-F_2,160_: 3.6218, *P*: 0.0252) total fresh weight (pseudo-F_1,160_: 52.845, *P*: 0.00001) as a covariate. No significant pairwise differences could be detected, but the differences between Le Perroquet and Westhoek (*P*: 0.0554) and between Westhoek and Ter Yde (*P*: 0.0601) were almost significant (Fig. 2, Table S3). Region turned out to be the only factor significantly affecting the aphids' population growth constant (pseudo-F_2,161_: 3.1898, *P*: 0.0427). A similar result (pseudo-F_2,160_: 3.2488, *P*: 0.0403) was obtained when the relative water content of the shoot was modelled as a covariate (pseudo-F_2,160_: 18.729, *P*: 0.00007). Aphid populations on plants from Ter Yde were characterised by stronger exponential growth, short generation times and larger maximum population sizes (Fig. 2). In all final models for aphid population parameters, variances did not significantly differ between groups (Levene's test, *P*>0.05).

In contrast to the plants they lived on, *S. rufula* aphids seemed not to be affected by the presence or nature of the biota in the soil. On the other hand, all tested population parameters significantly differed between regions where soil was collected. Since no interaction with inoculum was significant, some abiotic difference between the three dune regions must have been responsible for the observed differences. Therefore, we conclude that aphids are more affected by the abiotic properties of the soil than by the biotic soil component.

### Soil

Although most of the measured soil parameters differed significantly according to one or more treatment factors, none of the observed patterns suggested a logical causal link to the effects of the soil treatments on plant and/or aphid characteristics. Details of the differences in soil parameters between treatment soil mixtures are given in Text S1, Fig. S1, Fig. S2, Fig. S3, Table S1 and Table S2.

## Discussion

### Plants

The growth of *A. arenaria* seedlings was clearly more affected by the biotic than by the abiotic component of the soil they were planted in. There was no effect of the origin of the sterile soil part – dynamic or stabilised dune – on any of the tested plant characteristics. However, the effects of soil biota on biomass production were not consistent across regions.

We collected soils from dynamic dunes in winter, the period when most sand accretion occurs and plants have not yet developed roots into the newest layer [28]. Therefore the nutrients present in the fresh top layer of sand could not yet have been depleted by plants in the field. Since we could not detect any difference in plant performance between sterile soils from dynamic and stabilised dunes, the hypothesis that the decline of *A. arenaria* is due to the decreased availability of nutrients could not be confirmed in our study. The observation that biomass production was reduced by soil-borne biota from the rhizosphere of vital as well as degenerated plants is in accordance with previous work [25], [27]. However, this result was only confirmed for soils from Ter Yde. One explanation is that only Ter Yde soils contain organisms able to cause observable reductions in biomass within a limited amount of time, either because they are more pathogenic or because they have a higher proliferation rate. For example, some studies have demonstrated that certain root-feeding nematode species do not significantly affect the biomass of *A. arenaria* [30], [33], [34], [44]. Moreover, in a study using a comparable experimental setup [7], we found no effect of the extracted nematode community of Westhoek dune soils on either root or shoot biomass of *A. arenaria* seedlings. A second explanation might be that soils from Ter Yde contain relatively less organisms that are beneficial to the plant, such as mycorrhizae, or able to control plant antagonists, such as microbes and microarthropods. On the other hand, plants on sterile soil from Ter Yde produced significantly more biomass than those grown on sterile soil from Westhoek. This implies that some other, abiotic soil factor is limiting plant growth on Westhoek soils. Our experimental setup does not allow determining whether or not this abiotic effect is overruling a potential effect of soil biota as observed on Ter Yde soils. None of the measured soil properties was, however, indicative of a difference between Ter Yde and the other regions that could explain the observed pattern (see Text S1, Fig. S1, Fig. S2, Fig. S3, Table S1 and Table S2).

The proportion between root and shoot mass was not affected by the origin of the sterilised soil part, while plants inoculated with dynamic dune biota had a higher relative amount of roots. Since this effect was equal across the three regions, it seems that dynamic dunes, where *A. arenaria* thrives best, generally harbour soil organisms that affect the allocation of resources to different plant parts. Several studies have suggested that the windblown sand enables the plant to replace its old roots, hence increasing the ability to extract the necessary nutrients [20], [23]. Our results demonstrate that even in young seedlings, the formation of roots is increased in the presence of soil biota specific for the dune stages where sand accretion occurs. Interestingly, biota of later successional soils did not exert this effect. Relative root mass of plants grown on soil inoculated with stabilised dune biota was equal to that of plants grown on sterile soil. One explanation might be that the soil sampled at dynamic dune sites contains a lower total abundance of root antagonists than soil from stabilised dunes. There is evidence that at low densities, root-feeding nematodes can cause an enhanced translocation of photosynthate to roots, ultimately leading to an increase in root biomass [45], [46]. The positive effect on relative root mass of soil biota, at the low abundances typical of dynamic dunes, might thus have disappeared as densities increased under stabilising conditions. A second possibility is that a difference in the identity, rather than the abundance, between the soil biota from dynamic and stabilised dunes caused the observed difference in relative root mass. In a study on the nematode community along a sand dune succession in Scotland, Wall et al. [47] demonstrated a shift in species composition between foredunes on the one hand and yellow dunes and grey dunes on the other. They further found that both total and plant-feeding nematode abundance increased along the successional gradient, indicating that both proposed mechanisms could be operating synergistically. Both abundance and diversity of arbuscular mycorrhizal fungi were moreover found to be higher in isolates from vital than from degenerate stands of *A. arenaria* in coastal dunes of the Netherlands [48]. In dynamic dunes, the lower net effect of plant antagonists might thus be further mitigated by the higher abundance of these fungi, since they are mutualistic to the plant.

### Aphids

Contrary to our hypotheses, none of the tested aphid population characteristics differed between soils or inocula from dynamic and stabilised dunes. However, differences were detected in aphid population size, exponential growth constant and individual generation time between soils from the three regions along the coast. Aphids displayed the shortest generation times, steepest exponential growth and largest final population sizes on plants grown on soils from Ter Yde. These results did not change after correcting for the most significant correlations with measured plant characteristics, indicating that they are due to changes in the host plant of a fine-scale physiological nature. If these effects were due to differences in soil biota, they would have been detected for inoculated soils only, leading to a region x inoculum interaction. The observation that differences were independent of the biotic state of the soil, provides indirect proof that they must have been due to differences in one or more abiotic properties of the soils from the three regions. Although significant main effects of region on soil pH, % organic matter, % CaCO_3_ and plant available P were detected (see Text S1, Fig. S2, Fig. S3, Table S1 and Table S2), the inspection of significant interaction terms with soil and inoculum demonstrated that none of these soil parameters would logically explain the observed differences in aphid population properties across regions. For example, the higher amount of plant available P in Ter Yde soils might seem to explain why aphids performed better on plants grown on soils from this region. However, when regressing the maximum number of aphids on amount of P within each region (data not shown), contrasting results are obtained for each region, indicating that the relation between P and aphid population size does not hold. Although N would make a plausible predictor of aphid performance, no differences in NO_3_-N or NH_4_-N content could be detected between soils from different regions. It thus seems that in our system, aphids were not affected by any of the measured soil characteristics, not even by N or P availability, although these are the two elements generally considered to be limiting for herbivorous insects [3], [4]. It is possible that differences in the content of some other (micro)nutrient or in some physical property of the soil, e.g. pore size, caused the observed effects of region on aphids, especially given the subtle nature of the plant features that caused these effects.

The observation that soil biota did not affect aphid performance, although they did affect plant performance to some extent, is not in line with the bulk of literature documenting on the interactions between below- and aboveground biota [5]–[9]. However, some effects of soil biota on aboveground herbivores have been shown to only become apparent under particular levels of drought [49], [50]. Since our plants were watered ad libitum, soil biota might have been unable to impose stress on plants that would elicit a response of the aphids. Interestingly, in a similar laboratory setup, we have previously demonstrated a negative effect of the community of root-feeding nematodes on the population size of *S. rufula* [7]. However, the nematodes in the cited study were obtained by extraction from large quantities of roots, and subsequently concentrated to rather high densities before inoculation. Here we chose to use a complete soil inoculum, in order to address the more general question of the relative importance of the biotic soil component as such. Therefore, the volume of unsterile soil had to be small compared to the sterilised bulk part, in order not to quantitatively change the abiotic properties of the inoculated soils. The number of nematodes in such a small soil volume, especially root-feeding ones, is probably too low to cause a similar negative effect. However, the densities of biota applied here probably reflect the field situation more accurately. This is further supported by the lack of correlation between root-feeding nematode and aphid abundances in the field survey of the cited study.

### Implications of spatial variation

The fact that all the aphid population variables differed according to the soil's region of origin, but not according to the successional state of the dune soil or the biota present in that soil, emphasises the importance of replication at larger spatial scales. If our study would only have focused on one particular dune system, we would probably have concluded that no single aspect of the soil affected the development of aphid populations. As for the plant characteristics, the total dry biomass of plants only differed according to soil biota for soils from the Ter Yde dune area. By replicating our setup across dune areas, it became clear that this effect of biota is not a general phenomenon. The positive effect of dynamic dune biota on relative root biomass on the other hand turned out to hold true for each of the three dune areas, thereby proving the generality of this relationship. The specificity of some effects of biota for certain locations was to be expected since the spatial distribution of soil organisms is generally heterogeneous. This implies that at different locations, different species assemblages of soil biota occur, as for example demonstrated for root-feeding nematode species of the genus *Pratylenchus* associated with *A. arenaria* [51]. Given this variability in the universality and/or magnitude of the observed effects across spatially separated systems, spatial replication in future studies of above-belowground interactions, and of ecology in general, is needed to provide a better understanding of the generality of the ongoing processes.

### Conclusions

In order to unravel the relative effects of the abiotic and biotic components of soil on plant and insect herbivore performance, we chose *A. arenaria* as a model species because of its specific ecology. The decline of this species under decreasing sand dynamics has been attributed either to soil biota or to abiotic soil properties in several studies over the past decades. Our study did not yield convincing evidence of abiotic soil effects on plant performance, while soil biota did affect plant traits, in accordance with the escape hypothesis, although not all effects were apparent in all of the investigated dune areas. Even though the potential of soil biota to affect aphid population dynamics has previously been demonstrated in this system, this study could not detect any effect of soil biota on the performance of aphids. Differences of abiotic nature between soils from the three dune areas however affected all of the tested aphid population parameters. Our results therefore suggest that the biotic soil component is more important than the abiotic component in affecting plant performance, while the opposite holds true for the insect herbivore.

## Supporting Information

Text S1 Differences in soil characteristics between different treatment soils, according to abiotic and biotic soil component and soil region of origin.
(0.03 MB DOC)

Table S1 Results of the final permutational ANOVA models of the different soil parameters, obtained after a stepwise backward selection procedure. R: region where soil was collected: Westhoek, Ter Yde or Le Perroquet. S: sterile, abiotic component of the soil: dynamic dunes or stabilised dunes. I: unsterile, biotic soil inoculum: none, dynamic dunes or stabilised dunes. No significant effects were detected for the percentage moisture and NH4-N (mg/kg) of the soils.
(0.05 MB DOC)

Table S2 Results of pairwise comparisons among levels of factors that significantly affected the soil parameters in the final permutational ANOVA models. Comparisons were made by means of permutational t-tests. When the number of unique permutations was lower than 100, Monte Carlo sampling was used to obtain reliable P-values. Region - PE: Le Perroquet, WE: Westhoek, TY: Ter Yde. Soil - D: sterile soil component of dynamic dune, S: sterile soil component of stabilised dune. Inoculum - /: no inoculum, d: dynamic dune biota, s: stabilised dune biota.
(0.28 MB DOC)

Table S3 Results of pairwise comparisons among levels of factors that significantly affected plant and aphid characteristics in the final permutational ANOVA models. Comparisons were made by means of permutational t-tests. When the number of unique permutations was lower than 100, Monte Carlo sampling was used to obtain reliable P-values. Region - PE: Le Perroquet, WE: Westhoek, TY: Ter Yde. Inoculum - /: no inoculum, d: dynamic dune biota, s: stabilised dune biota.
(0.09 MB DOC)

Figure S1 Differences in NO3-N content of treatment soils with different unsterile inocula (mean + SE). Significant pairwise differences are indicated by different letters above the bars (P < 0.05). Inoculum - /: no inoculum, d: dynamic dune biota, s: stabilised dune biota.
(0.05 MB TIF)

Figure S2 Differences in soil parameters of treatment soils according to region and abiotic soil component (mean + SE). A) Percentage CaCO3. B) pH-KCl. C) Percentage organic matter per dry matter. Significant pairwise differences are indicated by different letters above the bars (P < 0.05). Region - PE: Le Perroquet, WE: Westhoek, TY: Ter Yde. Soil - D: sterile soil component of dynamic dune, S: sterile soil component of stabilised dune.
(0.12 MB TIF)

Figure S3 Differences in plant available P of treatment soils according to region, abiotic soil component and unsterile soil inoculum (mean + SE). Significant pairwise differences are indicated by different letters above the bars (P < 0.05). Region - PE: Le Perroquet, WE: Westhoek, TY: Ter Yde. Soil - D: sterile soil component of dynamic dune, S: sterile soil component of stabilised dune. Inoculum - /: no inoculum, d: dynamic dune biota, s: stabilised dune biota.
(0.17 MB TIF)
