# Supplementary material for: Relative Importance of Biotic and Abiotic Soil Components to Plant Growth and Insect Herbivore Population Dynamics
Source: PLoS One. 2010 Sep 23;5(9):e12937. doi: 10.1371/journal.pone.0012937 (PMC2944872; doi:10.1371/journal.pone.0012937)
Supplement: Text S1 — Differences in soil characteristics between different treatment soils, according to abiotic and biotic soil component and soil region of origin. (0.03 MB DOC) [file pone.0012937.s001.doc]

Mineral nitrogen content (both NO3-N and NH4-N) was extracted in a 1:5 soil to 1 M KCl solution according to ISO 14256-2 and measured with a Foss Fiastar 5000 continuous flow analyser. Soil moisture content (expressed on fresh weight) was determined as the weight loss at 105°C. The pH was measured in a 1M KCl solution according to ISO 10390. Inorganic C (IC) was measured with a Skalar Primacs SLC TOC-analyser. This result was converted to CaCO_3_ content. Organic matter content in the soil was estimated according to EN 13039. Plant available P was determined by the method of Olsen et al. (1954).

The amount of NO_3_-N significantly differed between soils with a different inoculum (Fig S1, Table S1). Fully sterile soils contained less NO_3_-N than soils with either of both unsterile inocula (Table S2). This indicates that soil fauna were able to increase the amount of soil nitrate. If anything, plants performed worse on inoculated soils. Therefore this increase in soil nitrate did not benefit plants in our study. The NH_4_-N content or moisture content did not significantly differ between any of the soil treatments. There was a significant interactive effect of abiotic soil and region on the percentage CaCO_3_ as well as on the percentage of organic matter and the pH (Fig S2, Table S1). Both at Le Perroquet and Ter Yde, soils from dynamic dunes had a higher CaCO_3_ content than soils from stabilised dunes, with the highest content found in dynamic dune soil from Ter Yde. The pH of the soil mixtures significantly differed according to an interaction between region and abiotic soil component (Fig S2, Table S1). Both at Le Perroquet and Westhoek, soils with a sterile component from dynamic dunes had a higher pH than stabilised dune soils (Table S2). Percentage of organic matter per dry matter significantly differed between different combinations of abiotic soil component and region (Fig S2, Table S1). Soils from Westhoek and Le Perroquet had a higher organic matter content when the abiotic soil component originated from stabilised dunes compared to soils with a sterile part from dynamic dunes, while the opposite was true for soils from Ter Yde (Table S2). The amount of plant available phosphorus differed according to a significant region x soil x inoculum interaction (Fig S3, Table S1, Table S2).
