# Supplementary material for: Relative Importance of Biotic and Abiotic Soil Components to Plant Growth and Insect Herbivore Population Dynamics
Source: PLoS One. 2010 Sep 23;5(9):e12937. doi: 10.1371/journal.pone.0012937 (PMC2944872; doi:10.1371/journal.pone.0012937)
Supplement: Table S1 — Results of the final permutational ANOVA models of the different soil parameters, obtained after a stepwise backward selection procedure. R: region where soil was collected: Westhoek, Ter Yde or Le Perroquet. S: sterile, abiotic component of the soil: dynamic dunes or stabilised dunes. I: unsterile, biotic soil inoculum: none, dynamic dunes or stabilised dunes. No significant effects were detected for the percentage moisture and NH4-N (mg/kg) of the soils. (0.05 MB DOC) [file pone.0012937.s002.doc]

| Source | df | SS | MS | pseudo-F | P | unique perms |
| --- | --- | --- | --- | --- | --- | --- |
| **NO3-N (mg/kg)** | | | | | | |
| I | 2, 51 | 2406.8 | 1203.4 | 5.7658 | 0.0057 | 73019 |
| **pH-KCl** | | | | | | |
| R | 2, 48 | 0.46815 | 0.23407 | 5.175 | 0.0077 | 95318 |
| S | 1, 48 | 1.1557 | 1.1557 | 25.552 | 0.00001 | 90408 |
| RxS | 2, 48 | 0.41926 | 0.20963 | 4.6346 | 0.0137 | 95441 |
| **% organic matter/dry matter** | | | | | | |
| R | 2, 48 | 0.1908 | 0.0954 | 20.786 | 0.00001 | 95511 |
| S | 1, 48 | 0.15254 | 0.15254 | 33.233 | 0.00001 | 90738 |
| RxS | 2, 48 | 0.45234 | 0.22617 | 49.276 | 0.00001 | 95355 |
| **% CaCO3** | | | | | | |
| R | 2, 48 | 9.9176 | 4.9588 | 11.846 | 0.0001 | 95378 |
| S | 1, 48 | 16.192 | 16.192 | 38.681 | 0.00001 | 90511 |
| RxS | 2, 48 | 8.9113 | 4.4557 | 10.644 | 0.0002 | 95384 |
| **Plant available P (mg/kg)** | | | | | | |
| R | 2, 36 | 40.193 | 20.097 | 161.67 | 0.00001 | 95425 |
| S | 1, 36 | 1.8297 | 1.8297 | 14.719 | 0.0004 | 90681 |
| I | 2, 36 | 0.0630 | 0.0315 | 0.25337 | 0.7804 | 95479 |
| RxS | 2, 36 | 15.603 | 7.8017 | 62.761 | 0.00001 | 95507 |
| RxI | 4, 36 | 0.13354 | 0.0334 | 0.26857 | 0.8978 | 95608 |
| SxI | 2, 36 | 1.4319 | 0.71594 | 5.7594 | 0.0062 | 95582 |
| RxSxI | 4, 36 | 1.9355 | 0.48387 | 3.8926 | 0.0092 | 95387 |
