# Supplementary material for: Relative Importance of Biotic and Abiotic Soil Components to Plant Growth and Insect Herbivore Population Dynamics
Source: PLoS One. 2010 Sep 23;5(9):e12937. doi: 10.1371/journal.pone.0012937 (PMC2944872; doi:10.1371/journal.pone.0012937)
Supplement: Table S2 — Results of pairwise comparisons among levels of factors that significantly affected the soil parameters in the final permutational ANOVA models. Comparisons were made by means of permutational t-tests. When the number of unique permutations was lower than 100, Monte Carlo sampling was used to obtain reliable P-values. Region - PE: Le Perroquet, WE: Westhoek, TY: Ter Yde. Soil - D: sterile soil component of dynamic dune, S: sterile soil component of stabilised dune. Inoculum - /: no inoculum, d: dynamic dune biota, s: stabilised dune biota. (0.28 MB DOC) [file pone.0012937.s003.doc]

| Groups | df | pseudo-t | *P* | unique perms | *P* (MC) |
| --- | --- | --- | --- | --- | --- |
| **NO3-N** | | | | | |
| / - d | 34 | 2.9549 | 0.0058 | 1471 | - |
| / - s | 34 | 2.8711 | 0.0075 | 1644 | - |
| d - s | 34 | 0.27735 | 0.7827 | 1370 | - |
| **pH-KCl** | | | | | |
| PE,D - PE,S | 16 | 2.7727 | 0.0081 | 19 | 0.0139 |
| PE,D - TY,D | 16 | 3.7811 | 0.0042 | 13 | 0.0017 |
| PE,D - TY,S | 16 | 3.8933 | 0.0018 | 15 | 0.0014 |
| PE,D - WE,D | 16 | 1.6557 | 0.1482 | 12 | 0.1185 |
| PE,D - WE,S | 16 | 13.252 | 0.00003 | 28 | 0.00001 |
| PE,S - TY,D | 16 | 1.0396 | 0.3942 | 17 | 0.3136 |
| PE,S - TY,S | 16 | 0.57932 | 0.6552 | 18 | 0.571 |
| PE,S - WE,D | 16 | 1.2689 | 0.2609 | 23 | 0.2237 |
| PE,S - WE,S | 16 | 2.3117 | 0.0448 | 21 | 0.0341 |
| TY,D - TY,S | 16 | 0.73521 | 0.5707 | 10 | 0.4747 |
| TY,D - WE,D | 16 | 0.55989 | 0.674 | 14 | 0.5833 |
| TY,D - WE,S | 16 | 7.3472 | 0.00005 | 20 | 0.00001 |
| TY,S - WE,D | 16 | 1.0398 | 0.3642 | 17 | 0.317 |
| TY,S - WE,S | 16 | 5.2507 | 0.0004 | 19 | 0.00006 |
| WE,D - WE,S | 16 | 5.0328 | 0.0009 | 25 | 0.0001 |
| **% organic matter/dry matter** | | | | | |
| PE,D - PE,S | 16 | 2.4771 | 0.0291 | 36 | 0.0246 |
| PE,D - TY,D | 16 | 0.76964 | 0.5137 | 37 | 0.4547 |
| PE,D - TY,S | 16 | 1.9505 | 0.0746 | 57 | 0.0687 |
| PE,D - WE,D | 16 | 1.7165 | 0.1137 | 50 | 0.1063 |
| PE,D - WE,S | 16 | 12.646 | 0.00004 | 117 | 0.00001 |
| PE,S - TY,D | 16 | 0.98263 | 0.4352 | 31 | 0.342 |
| PE,S - TY,S | 16 | 3.5711 | 0.0051 | 67 | 0.0027 |
| PE,S - WE,D | 16 | 3.4101 | 0.0048 | 61 | 0.0033 |
| PE,S - WE,S | 16 | 10.271 | 0.00001 | 98 | 0.00001 |
| TY,D - TY,S | 16 | 2.2209 | 0.0462 | 63 | 0.0411 |
| TY,D - WE,D | 16 | 2.0246 | 0.0676 | 61 | 0.0603 |
| TY,D - WE,S | 16 | 8.5979 | 0.00004 | 104 | 0.00001 |
| TY,S - WE,D | 16 | 0.25245 | 0.8207 | 60 | 0.804 |
| TY,S - WE,S | 16 | 10.624 | 0.00004 | 146 | 0.00001 |
| WE,D - WE,S | 16 | 10.75 | 0.00004 | 141 | 0.00001 |
| **% CaCO_3_** | | | | | |
| PE,D - PE,S | 16 | 2.9779 | 0.0082 | 394 | 0.0089 |
| PE,D - TY,D | 16 | 3.2156 | 0.007 | 426 | 0.0051 |
| PE,D - TY,S | 16 | 2.8422 | 0.0114 | 446 | 0.0115 |
| PE,D - WE,D | 16 | 2.8033 | 0.0138 | 370 | 0.0128 |
| PE,D - WE,S | 16 | 3.6072 | 0.0025 | 359 | 0.0025 |
| PE,S - TY,D | 16 | 6.4534 | 0.0002 | 591 | 0.00002 |
| PE,S - TY,S | 16 | 0.1867 | 0.8578 | 313 | 0.8553 |
| PE,S - WE,D | 16 | 0.28751 | 0.7834 | 273 | 0.7764 |
| PE,S - WE,S | 16 | 0.50859 | 0.6173 | 249 | 0.6168 |
| TY,D - TY,S | 16 | 6.0123 | 0.0003 | 643 | 0.00004 |
| TY,D - WE,D | 16 | 6.3542 | 0.0003 | 596 | 0.00002 |
| TY,D - WE,S | 16 | 7.2643 | 0.0001 | 596 | 0.00002 |
| TY,S - WE,D | 16 | 0.43833 | 0.6692 | 332 | 0.6685 |
| TY,S - WE,S | 16 | 0.2284 | 0.8302 | 294 | 0.8211 |
| WE,D - WE,S | 16 | 0.8483 | 0.4114 | 259 | 0.4106 |
| **Plant available P** | | | | | |
| PE,D,/ - PE,D,d | 4 | 1.1543 | 0.4013 | 10 | 0.313 |
| PE,D,/ - PE,D,s | 4 | 0.43329 | 0.7016 | 8 | 0.6884 |
| PE,D,/ - PE,S,/ | 4 | 5.7183 | 0.1017 | 10 | 0.0046 |
| PE,D,/ - PE,S,d | 4 | 8.5208 | 0.1001 | 10 | 0.0011 |
| PE,D,/ - PE,S,s | 4 | 5.0964 | 0.1009 | 10 | 0.0066 |
| PE,D,/ - TY,D,/ | 4 | 8.9317 | 0.0995 | 10 | 0.001 |
| PE,D,/ - TY,D,d | 4 | 7.4831 | 0.0998 | 10 | 0.0018 |
| PE,D,/ - TY,D,s | 4 | 20.575 | 0.0997 | 10 | 0.00001 |
| PE,D,/ - TY,S,/ | 4 | 5.5385 | 0.1005 | 10 | 0.005 |
| PE,D,/ - TY,S,d | 4 | 12.823 | 0.0995 | 10 | 0.0001 |
| PE,D,/ - TY,S,s | 4 | 18.033 | 0.1006 | 9 | 0.0001 |
| PE,D,/ - WE,D,/ | 4 | 4.222 | 0.0995 | 10 | 0.0133 |
| PE,D,/ - WE,D,d | 4 | 7.6896 | 0.0988 | 10 | 0.0016 |
| PE,D,/ - WE,D,s | 4 | 6.5463 | 0.1004 | 10 | 0.0028 |
| PE,D,/ - WE,S,/ | 4 | 20.019 | 0.0994 | 10 | 0.00002 |
| PE,D,/ - WE,S,d | 4 | 21.5 | 0.1012 | 10 | 0.00003 |
| PE,D,/ - WE,S,s | 4 | 6.8147 | 0.0997 | 10 | 0.0026 |
| PE,D,d - PE,D,s | 4 | 1.6207 | 0.303 | 10 | 0.1809 |
| PE,D,d - PE,S,/ | 4 | 4.0989 | 0.1 | 10 | 0.0147 |
| PE,D,d - PE,S,d | 4 | 4.6323 | 0.0999 | 10 | 0.0097 |
| PE,D,d - PE,S,s | 4 | 3.1384 | 0.1007 | 10 | 0.0345 |
| PE,D,d - TY,D,/ | 4 | 7.8804 | 0.1002 | 10 | 0.0015 |
| PE,D,d - TY,D,d | 4 | 6.9547 | 0.0997 | 10 | 0.0023 |
| PE,D,d - TY,D,s | 4 | 17.144 | 0.0988 | 9 | 0.00007 |
| PE,D,d - TY,S,/ | 4 | 5.0503 | 0.1004 | 10 | 0.0073 |
| PE,D,d - TY,S,d | 4 | 9.8311 | 0.1 | 10 | 0.0008 |
| PE,D,d - TY,S,s | 4 | 11.152 | 0.1002 | 10 | 0.0004 |
| PE,D,d - WE,D,/ | 4 | 3.222 | 0.0998 | 10 | 0.032 |
| PE,D,d - WE,D,d | 4 | 4.7752 | 0.0988 | 10 | 0.009 |
| PE,D,d - WE,D,s | 4 | 5.0685 | 0.0997 | 10 | 0.0067 |
| PE,D,d - WE,S,/ | 4 | 14.747 | 0.098 | 10 | 0.0001 |
| PE,D,d - WE,S,d | 4 | 16.243 | 0.101 | 10 | 0.00007 |
| PE,D,d - WE,S,s | 4 | 6.114 | 0.1002 | 10 | 0.0035 |
| PE,D,s - PE,S,/ | 4 | 6.5889 | 0.1002 | 9 | 0.0026 |
| PE,D,s - PE,S,d | 4 | 14.473 | 0.0998 | 10 | 0.00007 |
| PE,D,s - PE,S,s | 4 | 6.3661 | 0.0996 | 10 | 0.0031 |
| PE,D,s - TY,D,/ | 4 | 9.3939 | 0.1003 | 10 | 0.0008 |
| PE,D,s - TY,D,d | 4 | 7.6696 | 0.0998 | 10 | 0.0017 |
| PE,D,s - TY,D,s | 4 | 22.96 | 0.0997 | 10 | 0.00002 |
| PE,D,s - TY,S,/ | 4 | 5.7001 | 0.1013 | 10 | 0.0047 |
| PE,D,s - TY,S,d | 4 | 15.009 | 0.1024 | 10 | 0.0001 |
| PE,D,s - TY,S,s | 4 | 30.86 | 0.1006 | 10 | 0.00001 |
| PE,D,s - WE,D,/ | 4 | 4.6247 | 0.1005 | 10 | 0.0096 |
| PE,D,s - WE,D,d | 4 | 10.346 | 0.0986 | 9 | 0.0005 |
| PE,D,s - WE,D,s | 4 | 7.2912 | 0.0994 | 10 | 0.0018 |
| PE,D,s - WE,S,/ | 4 | 25.222 | 0.0989 | 10 | 0.00001 |
| PE,D,s - WE,S,d | 4 | 26.419 | 0.0994 | 10 | 0.00003 |
| PE,D,s - WE,S,s | 4 | 7.0774 | 0.0985 | 10 | 0.002 |
| PE,S,/ - PE,S,d | 4 | 1.226 | 0.4032 | 10 | 0.2865 |
| PE,S,/ - PE,S,s | 4 | 1.5132 | 0.2003 | 9 | 0.2031 |
| PE,S,/ - TY,D,/ | 4 | 5.0391 | 0.0999 | 10 | 0.007 |
| PE,S,/ - TY,D,d | 4 | 5.1858 | 0.0997 | 10 | 0.0064 |
| PE,S,/ - TY,D,s | 4 | 12.197 | 0.1013 | 10 | 0.0003 |
| PE,S,/ - TY,S,/ | 4 | 3.3069 | 0.099 | 10 | 0.0286 |
| PE,S,/ - TY,S,d | 4 | 4.8674 | 0.1007 | 10 | 0.008 |
| PE,S,/ - TY,S,s | 4 | 4.3681 | 0.0996 | 10 | 0.0115 |
| PE,S,/ - WE,D,/ | 4 | 0.0654 | 1 | 10 | 0.9514 |
| PE,S,/ - WE,D,d | 4 | 0.5167 | 0.7017 | 7 | 0.6303 |
| PE,S,/ - WE,D,s | 4 | 1.2863 | 0.4001 | 10 | 0.2668 |
| PE,S,/ - WE,S,/ | 4 | 8.619 | 0.1001 | 10 | 0.0009 |
| PE,S,/ - WE,S,d | 4 | 10.155 | 0.0996 | 10 | 0.0003 |
| PE,S,/ - WE,S,s | 4 | 3.8775 | 0.1002 | 10 | 0.0184 |
| PE,S,d - PE,S,s | 4 | 0.79152 | 0.4997 | 10 | 0.4722 |
| PE,S,d - TY,D,/ | 4 | 6.3816 | 0.1009 | 10 | 0.0032 |
| PE,S,d - TY,D,d | 4 | 5.8504 | 0.0987 | 10 | 0.0047 |
| PE,S,d - TY,D,S | 4 | 17.866 | 0.101 | 10 | 0.00005 |
| PE,S,d - TY,S,/ | 4 | 3.8794 | 0.0999 | 10 | 0.0178 |
| PE,S,d - TY,S,d | 4 | 8.7825 | 0.0995 | 10 | 0.0009 |
| PE,S,d - TY,S,s | 4 | 17.5 | 0.1017 | 9 | 0.00006 |
| PE,S,d - WE,D,/ | 4 | 0.91818 | 0.5016 | 10 | 0.411 |
| PE,S,d - WE,D,d | 4 | 1.16 | 0.4018 | 10 | 0.3106 |
| PE,S,d - WE,D,s | 4 | 2.6997 | 0.1002 | 10 | 0.0515 |
| PE,S,d - WE,S,/ | 4 | 17.408 | 0.1007 | 10 | 0.00004 |
| PE,S,d - WE,S,d | 4 | 19.143 | 0.0994 | 9 | 0.0001 |
| PE,S,d - WE,S,s | 4 | 4.7234 | 0.1009 | 10 | 0.0092 |
| PE,S,s - TY,D,/ | 4 | 6.2915 | 0.1011 | 10 | 0.0034 |
| PE,S,s - TY,D,d | 4 | 5.9036 | 0.1005 | 10 | 0.0044 |
| PE,S,s - TY,D,s | 4 | 15.425 | 0.1005 | 10 | 0.0001 |
| PE,S,s - TY,S,/ | 4 | 3.9799 | 0.099 | 10 | 0.0167 |
| PE,S,s - TY,S,d | 4 | 7.435 | 0.1005 | 9 | 0.0018 |
| PE,S,s - TY,S,s | 4 | 8.3593 | 0.1005 | 10 | 0.0012 |
| PE,S,s - WE,D,/ | 4 | 1.204 | 0.2993 | 10 | 0.2961 |
| PE,S,s - WE,D,d | 4 | 1.4297 | 0.3016 | 10 | 0.2262 |
| PE,S,s - WE,D,s | 4 | 2.7866 | 0.1009 | 9 | 0.049 |
| PE,S,s - WE,S,/ | 4 | 12.64 | 0.1002 | 10 | 0.0003 |
| PE,S,s - WE,S,d | 4 | 14.315 | 0.1004 | 10 | 0.0001 |
| PE,S,s - WE,S,s | 4 | 4.7846 | 0.0992 | 10 | 0.0091 |
| TY,D,/ - TY,D,d | 4 | 1.7416 | 0.3 | 10 | 0.1555 |
| TY,D,/ - TY,D,s | 4 | 3.2975 | 0.0988 | 10 | 0.0293 |
| TY,D,/ - TY,S,/ | 4 | 0.0460 | 1 | 10 | 0.9661 |
| TY,D,/ - TY,S,d | 4 | 2.0798 | 0.1998 | 10 | 0.1071 |
| TY,D,/ - TY,S,s | 4 | 3.2745 | 0.1018 | 10 | 0.031 |
| TY,D,/ - WE,D,/ | 4 | 4.4095 | 0.0987 | 10 | 0.012 |
| TY,D,/ - WE,D,d | 4 | 5.8271 | 0.0991 | 10 | 0.0046 |
| TY,D,/ - WE,D,s | 4 | 3.9109 | 0.1006 | 10 | 0.0166 |
| TY,D,/ - WE,S,/ | 4 | 0.2193 | 0.9008 | 9 | 0.8344 |
| TY,D,/ - WE,S,d | 4 | 0.85723 | 0.5993 | 9 | 0.4389 |
| TY,D,/ - WE,S,s | 4 | 0.18853 | 0.8987 | 10 | 0.8615 |
| TY,D,d - TY,D,s | 4 | 0.21067 | 0.8994 | 10 | 0.846 |
| TY,D,d - TY,S,/ | 4 | 1.3955 | 0.1992 | 10 | 0.2359 |
| TY,D,d - TY,S,d | 4 | 3.2725 | 0.1 | 9 | 0.0303 |
| TY,D,d - TY,S,s | 4 | 3.9848 | 0.1004 | 10 | 0.0161 |
| TY,D,d - WE,D,/ | 4 | 4.8749 | 0.1003 | 10 | 0.0083 |
| TY,D,d - WE,D,d | 4 | 5.5646 | 0.0988 | 10 | 0.0051 |
| TY,D,d - WE,D,s | 4 | 4.484 | 0.0999 | 10 | 0.0111 |
| TY,D,d - WE,S,/ | 4 | 2.1249 | 0.201 | 10 | 0.1028 |
| TY,D,d - WE,S,d | 4 | 1.4516 | 0.4 | 10 | 0.2211 |
| TY,D,d - WE,S,s | 4 | 1.7522 | 0.2021 | 10 | 0.153 |
| TY,D,s - TY,S,/ | 4 | 2.0834 | 0.2 | 10 | 0.1057 |
| TY,D,s - TY,S,d | 4 | 8.4474 | 0.1015 | 10 | 0.001 |
| TY,D,s - TY,S,s | 4 | 12.455 | 0.0994 | 10 | 0.0002 |
| TY,D,s - WE,D,/ | 4 | 9.6031 | 0.1013 | 10 | 0.0007 |
| TY,D,s - WE,D,d | 4 | 15.702 | 0.0999 | 10 | 0.00006 |
| TY,D,s - WE,D,s | 4 | 9.8912 | 0.0995 | 9 | 0.0006 |
| TY,D,s - WE,S,/ | 4 | 6.198 | 0.1005 | 10 | 0.0035 |
| TY,D,s - WE,S,d | 4 | 4.3262 | 0.1009 | 10 | 0.0122 |
| TY,D,s - WE,S,s | 4 | 2.9027 | 0.101 | 9 | 0.044 |
| TY,S,/ - TY,S,d | 4 | 1.3656 | 0.2986 | 10 | 0.2458 |
| TY,S,/ - TY,S,s | 4 | 2.0137 | 0.0988 | 9 | 0.1138 |
| TY,S,/ - WE,D,/ | 4 | 3.0965 | 0.1013 | 10 | 0.0363 |
| TY,S,/ - WE,D,d | 4 | 3.6165 | 0.0986 | 10 | 0.0226 |
| TY,S,/ - WE,D,s | 4 | 2.64 | 0.0998 | 10 | 0.0573 |
| TY,S,/ - WE,S,/ | 4 | 0.18757 | 0.7988 | 10 | 0.8582 |
| TY,S,/ - WE,S,d | 4 | 0.47853 | 0.699 | 10 | 0.6584 |
| TY,S,/ - WE,S,s | 4 | 0.18785 | 0.8003 | 10 | 0.8595 |
| TY,S,d - TY,S,s | 4 | 2.1638 | 0.1989 | 7 | 0.0969 |
| TY,S,d - WE,D,/ | 4 | 3.6518 | 0.0991 | 10 | 0.0219 |
| TY,S,d - WE,D,d | 4 | 7.0278 | 0.1001 | 10 | 0.0021 |
| TY,S,d - WE,D,s | 4 | 3.0058 | 0.1006 | 10 | 0.0398 |
| TY,S,d - WE,S,/ | 4 | 3.661 | 0.0997 | 10 | 0.0211 |
| TY,S,d - WE,S,d | 4 | 5.5224 | 0.1002 | 10 | 0.0055 |
| TY,S,d - WE,S,s | 4 | 1.4437 | 0.3012 | 10 | 0.2223 |
| TY,S,s - WE,D,/ | 4 | 2.9045 | 0.1016 | 10 | 0.0443 |
| TY,S,s - WE,D,d | 4 | 8.8403 | 0.0996 | 10 | 0.0011 |
| TY,S,s - WE,D,s | 4 | 2.0697 | 0.2016 | 10 | 0.1091 |
| TY,S,s - WE,S,/ | 4 | 8.7563 | 0.1005 | 10 | 0.0009 |
| TY,S,s - WE,S,d | 4 | 11.133 | 0.1003 | 10 | 0.0003 |
| TY,S,s - WE,S,s | 4 | 2.3056 | 0.1001 | 10 | 0.0825 |
| WE,D,/ - WE,D,d | 4 | 0.44573 | 0.8007 | 9 | 0.6801 |
| WE,D,/ - WE,D,s | 4 | 0.98803 | 0.5015 | 10 | 0.3814 |
| WE,D,/ - WE,S,/ | 4 | 6.2138 | 0.0999 | 10 | 0.0036 |
| WE,D,/ - WE,S,d | 4 | 7.4182 | 0.0992 | 9 | 0.0019 |
| WE,D,/ - WE,S,s | 4 | 3.5229 | 0.1007 | 10 | 0.0244 |
| WE,D,d - WE,D,s | 4 | 1.9911 | 0.1994 | 10 | 0.1161 |
| WE,D,d - WE,S,/ | 4 | 13.279 | 0.0991 | 10 | 0.0002 |
| WE,D,d - WE,S,d | 4 | 15.105 | 0.0994 | 10 | 0.0001 |
| WE,D,d - WE,S,s | 4 | 4.3525 | 0.0989 | 10 | 0.0119 |
| WE,D,s - WE,S,/ | 4 | 6.0988 | 0.0993 | 10 | 0.0038 |
| WE,D,s - WE,S,d | 4 | 7.5288 | 0.1006 | 10 | 0.0018 |
| WE,D,s - WE,S,s | 4 | 3.0119 | 0.0996 | 9 | 0.0389 |
| WE,S,/ - WE,S,d | 4 | 2.3413 | 0.2026 | 9 | 0.0799 |
| WE,S,/ - WE,S,s | 4 | 0.0578 | 0.9009 | 10 | 0.9563 |
| WE,S,d - WE,S,s | 4 | 0.90939 | 0.5009 | 9 | 0.4149 |
