# Supplementary material for: Relative Importance of Biotic and Abiotic Soil Components to Plant Growth and Insect Herbivore Population Dynamics
Source: PLoS One. 2010 Sep 23;5(9):e12937. doi: 10.1371/journal.pone.0012937 (PMC2944872; doi:10.1371/journal.pone.0012937)
Supplement: Table S3 — Results of pairwise comparisons among levels of factors that significantly affected plant and aphid characteristics in the final permutational ANOVA models. Comparisons were made by means of permutational t-tests. When the number of unique permutations was lower than 100, Monte Carlo sampling was used to obtain reliable P-values. Region - PE: Le Perroquet, WE: Westhoek, TY: Ter Yde. Inoculum - /: no inoculum, d: dynamic dune biota, s: stabilised dune biota. (0.09 MB DOC) [file pone.0012937.s004.doc]

| Groups | df | pseudo-t | *P* | unique perms | *P* (MC) |
| --- | --- | --- | --- | --- | --- |
| **Aphids** | | | | | |
| **Maximum # aphids** | | | | | |
| PE, TY | 108 | 3.5856 | 0.0007 | 618 | - |
| PE, WE | 106 | 1.525 | 0.132 | 272 | - |
| TY, WE | 108 | 2.2681 | 0.0254 | 582 | - |
| **Generation time** | | | | | |
| PE, TY | 108 | 0.08866 | 0.9654 | 68 | 0.9295 |
| PE, WE | 106 | 1.941 | 0.0476 | 55 | 0.0554 |
| TY, WE | 108 | 1.8968 | 0.0601 | 111 | 0.0601 |
| **Growth constant** | | | | | |
| PE, TY | 108 | 1.6854 | 0.0959 | 14490 | - |
| PE, WE | 106 | 0.82767 | 0.4157 | 9342 | - |
| TY, WE | 108 | 2.6324 | 0.0091 | 14962 | - |
| **Plants** | | | | | |
| **Total dry mass** | | | | | |
| PE,/ - PE,d | 21 | 0.79632 | 0.5023 | 2574 | - |
| PE,/ - PE,s | 25 | 0.44043 | 0.7 | 2980 | - |
| PE,/ - TY,/ | 23 | 1.3687 | 0.1859 | 4307 | - |
| PE,/ - TY,d | 23 | 2.5635 | 0.0071 | 3076 | - |
| PE,/ - TY,s | 21 | 0.71371 | 0.5073 | 1711 | - |
| PE,/ - WE,/ | 19 | 1.9281 | 0.0529 | 1494 | - |
| PE,/ - WE,d | 20 | 1.3289 | 0.2052 | 3001 | - |
| PE,/ - WE,s | 18 | 0.94134 | 0.3898 | 2794 | - |
| PE,d - PE,s | 24 | 0.65177 | 0.5231 | 1087 | - |
| PE,d - TY,/ | 22 | 2.3737 | 0.0266 | 3678 | - |
| PE,d - TY,d | 22 | 3.0053 | 0.0067 | 2046 | - |
| PE,d - TY,s | 20 | 0.0477 | 0.963 | 1205 | - |
| PE,d - WE,/ | 18 | 2.2495 | 0.0394 | 1828 | - |
| PE,d - WE,d | 19 | 0.99887 | 0.3279 | 2028 | - |
| PE,d - WE,s | 17 | 0.50239 | 0.6276 | 1839 | - |
| PE,s - TY,/ | 26 | 2.2135 | 0.0364 | 3832 | - |
| PE,s - TY,d | 26 | 3.4582 | 0.0023 | 1393 | - |
| PE,s - TY,s | 24 | 0.53076 | 0.601 | 1436 | - |
| PE,s - WE,/ | 22 | 2.6083 | 0.018 | 2313 | - |
| PE,s - WE,d | 23 | 1.5068 | 0.1424 | 2469 | - |
| PE,s - WE,s | 21 | 0.9657 | 0.3444 | 2285 | - |
| TY,/ - TY,d | 24 | 4.1195 | 0.0005 | 2341 | - |
| TY,/ - TY,s | 22 | 2.1235 | 0.0457 | 4118 | - |
| TY,/ - WE,/ | 20 | 3.2457 | 0.0048 | 2135 | - |
| TY,/ - WE,d | 21 | 2.7163 | 0.0141 | 4030 | - |
| TY,/ - WE,s | 19 | 2.1923 | 0.0415 | 3773 | - |
| TY,d - TY,s | 22 | 1.9901 | 0.0578 | 1391 | - |
| TY,d - WE,/ | 20 | 0.43026 | 0.6681 | 1758 | - |
| TY,d - WE,d | 21 | 1.3275 | 0.1961 | 2170 | - |
| TY,d - WE,s | 19 | 1.5695 | 0.1341 | 2017 | - |
| TY,s - WE,/ | 18 | 1.4171 | 0.1739 | 2479 | - |
| TY,s - WE,d | 19 | 0.68315 | 0.5002 | 2689 | - |
| TY,s - WE,s | 17 | 0.32852 | 0.7442 | 2499 | - |
| WE,/ - WE,d | 17 | 0.82191 | 0.4174 | 1947 | - |
| WE,/ - WE,s | 15 | 1.0662 | 0.3137 | 1775 | - |
| WE,d - WE,s | 16 | 0.31065 | 0.7597 | 2104 | - |
| **Root/total dry mass** | | | | |  |
| /, d | 66 | 2.3204 | 0.0082 | 9196 | - |
| /, s | 66 | 0.11853 | 0.9325 | 5781 | - |
| d, s | 66 | 2.5971 | 0.0008 | 7697 | - |
