# Supplementary figures and images for: Relative Importance of Biotic and Abiotic Soil Components to Plant Growth and Insect Herbivore Population Dynamics
Source: PLoS One. 2010 Sep 23;5(9):e12937. doi: 10.1371/journal.pone.0012937 (PMC2944872; doi:10.1371/journal.pone.0012937)

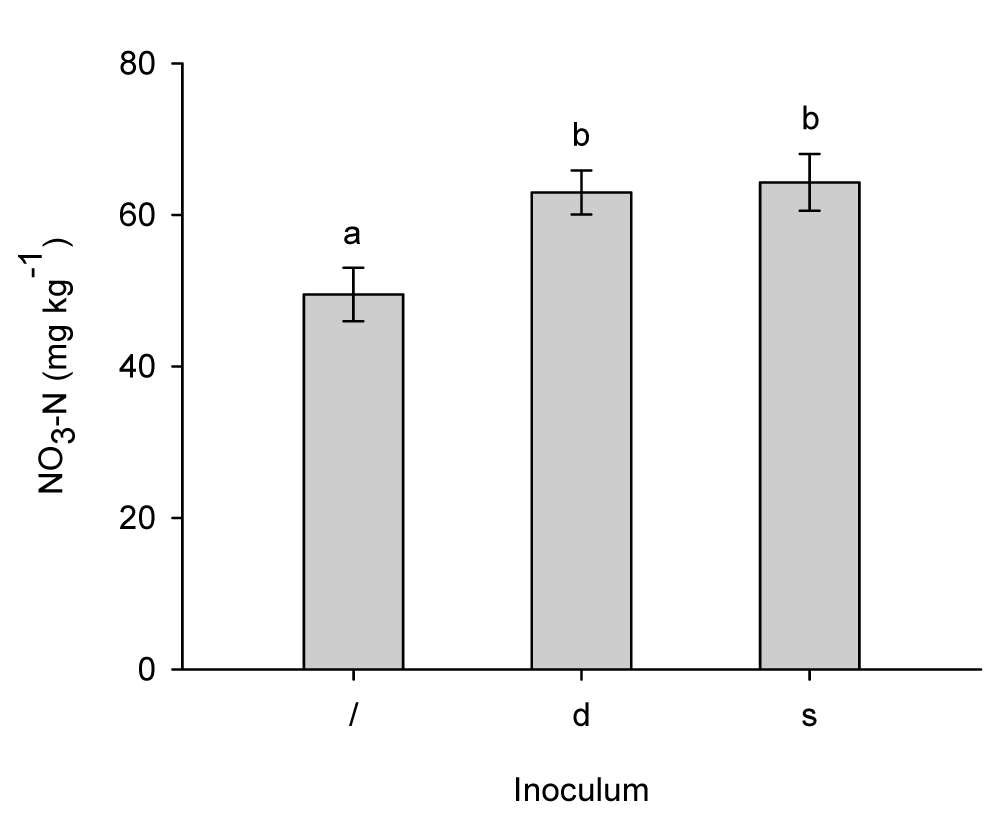

Supplement: Figure S1 — Differences in NO3-N content of treatment soils with different unsterile inocula (mean + SE). Significant pairwise differences are indicated by different letters above the bars (P < 0.05). Inoculum - /: no inoculum, d: dynamic dune biota, s: stabilised dune biota. (0.05 MB TIF) [file pone.0012937.s005.tif]

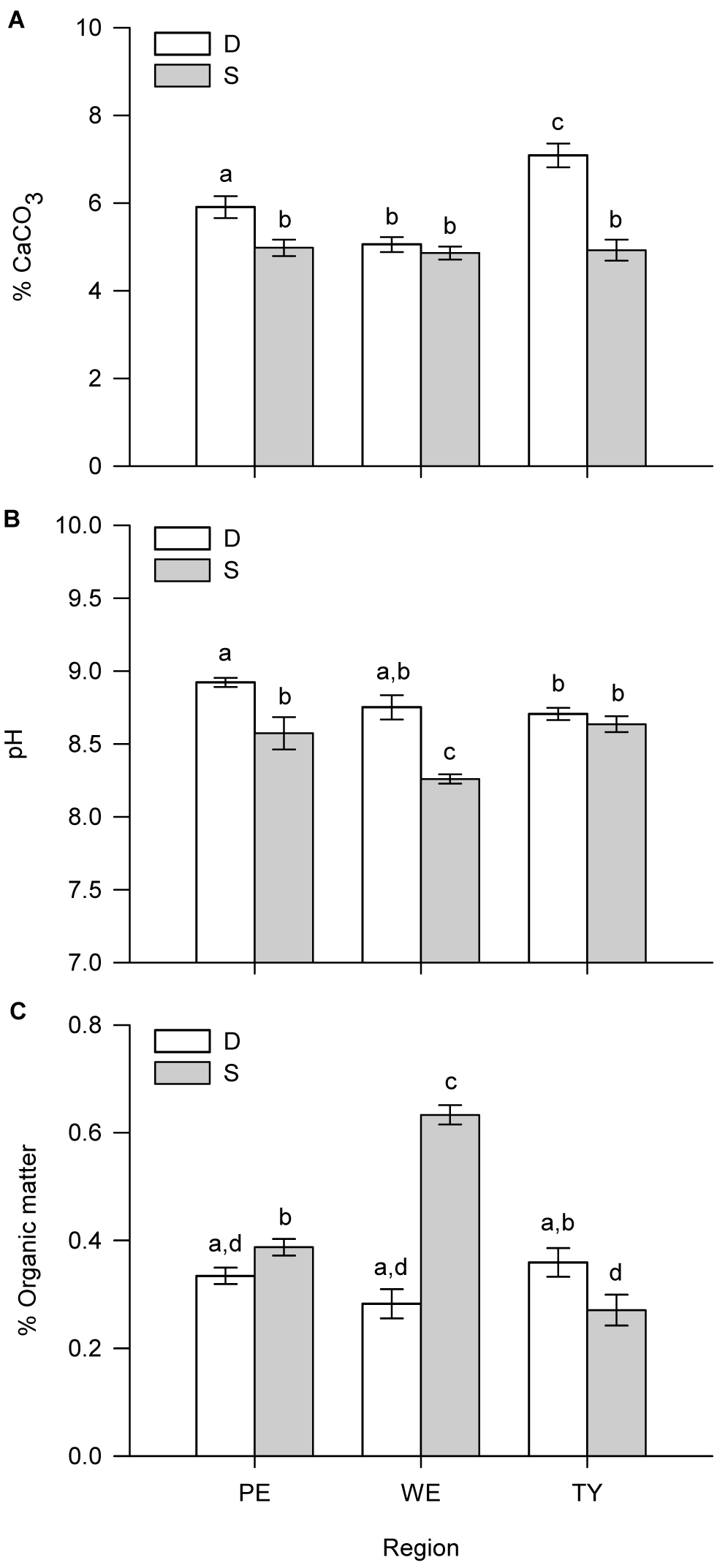

Supplement: Figure S2 — Differences in soil parameters of treatment soils according to region and abiotic soil component (mean + SE). A) Percentage CaCO3. B) pH-KCl. C) Percentage organic matter per dry matter. Significant pairwise differences are indicated by different letters above the bars (P < 0.05). Region - PE: Le Perroquet, WE: Westhoek, TY: Ter Yde. Soil - D: sterile soil component of dynamic dune, S: sterile soil component of stabilised dune. (0.12 MB TIF) [file pone.0012937.s006.tif]

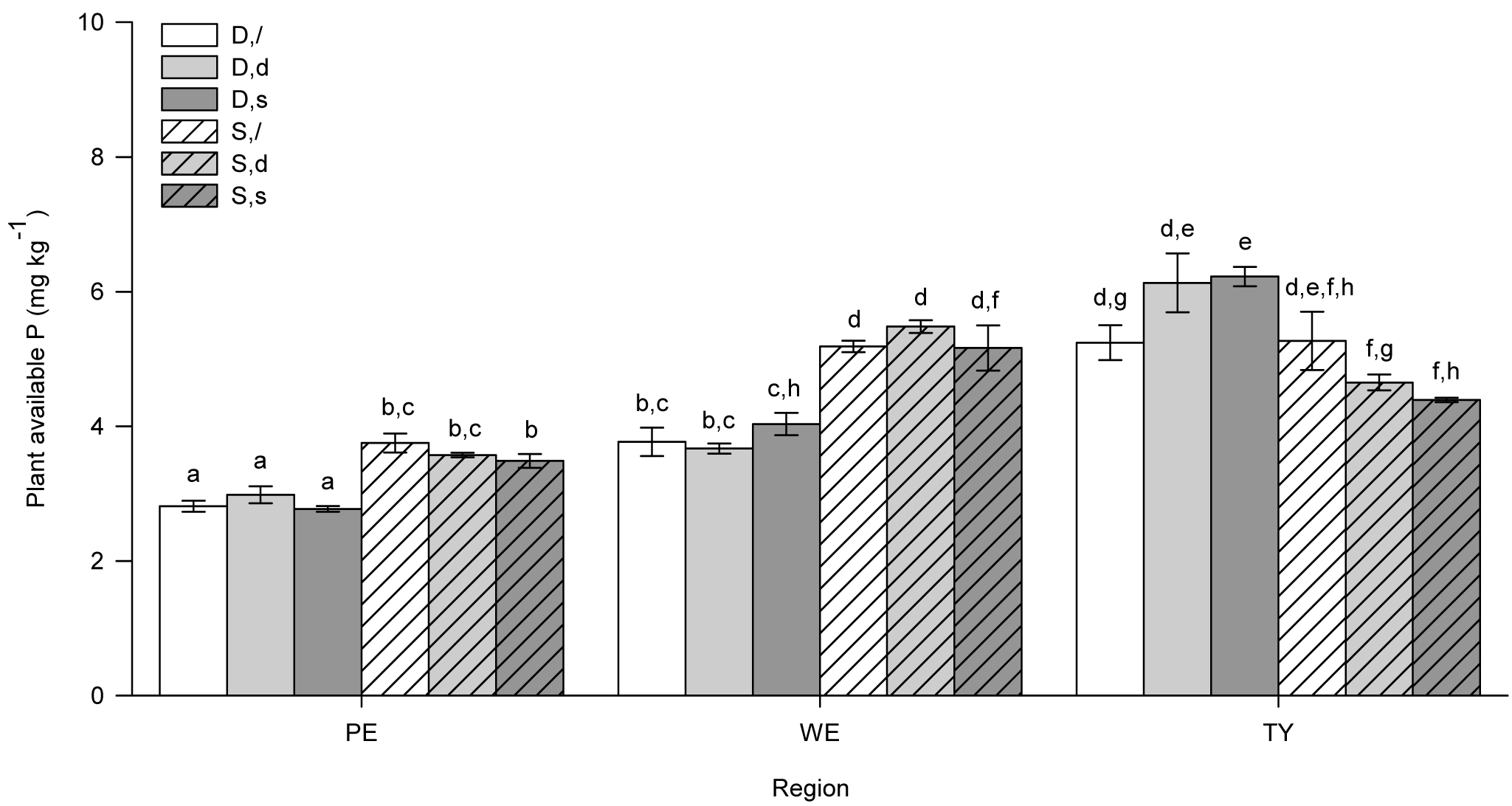

Supplement: Figure S3 — Differences in plant available P of treatment soils according to region, abiotic soil component and unsterile soil inoculum (mean + SE). Significant pairwise differences are indicated by different letters above the bars (P < 0.05). Region - PE: Le Perroquet, WE: Westhoek, TY: Ter Yde. Soil - D: sterile soil component of dynamic dune, S: sterile soil component of stabilised dune. Inoculum - /: no inoculum, d: dynamic dune biota, s: stabilised dune biota. (0.17 MB TIF) [file pone.0012937.s007.tif]
